# *Posidonia oceanica* (L.) Delile Extract Reduces Lipid Accumulation through Autophagy Activation in HepG2 Cells

**DOI:** 10.3390/ph14100969

**Published:** 2021-09-24

**Authors:** Marzia Vasarri, Emanuela Barletta, Donatella Degl’Innocenti

**Affiliations:** 1 Department of Experimental and Clinical Biomedical Sciences, University of Florence, Viale Morgagni 50, 50134 Florence, Italy; marzia.vasarri@unifi.it (M.V.); emanuela.barletta@unifi.it (E.B.); 2 Interuniversity Center of Marine Biology and Applied Ecology “G. Bacci” (CIBM), Viale N. Sauro 4, 57128 Livorno, Italy

**Keywords:** *Posidonia oceanica*, autophagy, lipid accumulation, NAFLD, herbal medicine

## Abstract

*Posidonia oceanica* (L.) Delile is a marine plant traditionally used as an herbal medicine for various health disorders. *P. oceanica* leaf extract (POE) has been shown to be a phytocomplex with cell-safe bioactivities, including the ability to trigger autophagy. Autophagy is a key pathway to counteract non-alcoholic fatty liver disease (NAFLD) by controlling the breakdown of lipid droplets in the liver. The aim of this study was to explore the ability of POE to trigger autophagy and reduce lipid accumulation in human hepatoma (HepG2) cells and then verify the possible link between the effect of POE on lipid reduction and autophagy activation. Expression levels of autophagy markers were monitored by the Western blot technique in POE-treated HepG2 cells, whereas the extent of lipid accumulation in HepG2 cells was assessed by Oil red O staining. Chloroquine (CQ), an autophagy inhibitor, was used to study the relationship between POE-induced autophagy and intracellular lipid accumulation. POE was found to stimulate an autophagy flux over time in HepG2 cells by lowering the phosphorylation state of ribosomal protein S6, increasing Beclin-1 and LC3-II levels, and decreasing p62 levels. By blocking autophagy with CQ, the effect of POE on intracellular lipid accumulation was clearly reversed, suggesting that the POE phytocomplex may reduce lipid accumulation in HepG2 cells by activating the autophagic process. This work indicates that *P. oceanica* may be considered as a promising molecule supplier to discover new natural approaches for the management of NAFLD.

## 1. Introduction

*Posidonica oceanica* (L.) Delile is a marine plant belonging to the Posidoniaceae family and the only species of Posidoniaceae endemic to the Mediterranean Sea [1]. Besides the extreme ecological importance of *P. oceanica* underwater meadows, the literature tells of a millenary relationship between humans and this marine plant. Some historical sources report that in Ancient Egypt, *P. oceanica* leaves were used as an herbal medicine for various health ailments, such as sore throat and skin problems [2], but also for the treatment of inflammation and irritation and as a remedy for acne, lower limb pain, and colitis [3]. More recently, a decoction of *P. oceanica* leaves was used as an herbal medicine against diabetes and hypertension by inhabitants of coastal areas in Western Anatolia [4]. In support of this, the literature describes antidiabetic and vasoprotective effects of a hydroalcoholic extract obtained from *P. oceanica* leaves in an in vivo animal model [4].

**Figure 1:**
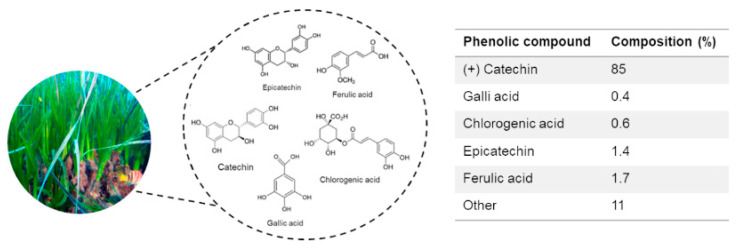
Phenolic composition with relative percentages of *P. oceanica* leaf extract (POE) obtained by UPLC analysis [5].

In recent years, our group has undertaken a series of in vitro and in vivo studies that for the first time shed light on the hitherto unexplored biological mechanisms underlying the bioactive properties of a hydroalcoholic *P. oceanica* leaf extract (POE). UPLC characterization analysis showed that POE consists of 88% phenolic compounds. Of these, about 85% were represented by (+) catechins and the remaining 4% by a mixture of gallic acid (0.4%), ferulic acid (1.7%), epicatechin (1.4%), and chlorogenic acid (0.6%). The small remaining fraction (11%) remained unknown/uncharacterized (Figure 1) [5]. Moreover, POE has been shown to be consistently effective as a phytocomplex at doses nontoxic to cells [5,6,7,8,9,10].

POE showed anti-inflammatory properties both in an in vitro cell model of lipopolysaccharide (LPS)-stimulated murine macrophages [8] and in different in vivo models of inflammatory pain induced by intraplantar injection of carrageenan, interleukin IL-1β, and formalin in CD-1 mice [9]. In addition, POE showed an inhibitory role on the in vitro protein glycation process by blocking the formation of advanced glycation end products (AGEs) [10], as well as the ability to inhibit the highly migratory phenotype of certain cell lines such as human fibrosarcoma HT1080 [5,6] and human neuroblastoma SH-SY5Y [7]. Further, our investigation of the mechanism of action underlying its anti-migratory role revealed that POE acts by activating a transient autophagy flux [6].

Autophagy is an evolutionarily conserved intracellular degradative process that regulates metabolism and maintains cellular homeostasis by removing aggregated, damaged and/or misfolded proteins, and damaged organelles through cytosolic sequestration and subsequent lysosomal degradation [11]. In the light of this, it is known that a deficiency in autophagy could contribute to the development or progression of several disease conditions, including non-alcoholic fatty liver disease (NAFLD) [12,13]. NAFLD is the most common liver disease in Western countries and is defined as evidence of hepatic steatosis without any cause of secondary hepatic fat accumulation, such as alcohol abuse, use of steatogenic drugs, or inherited disorders [14]. In NAFLD, cells are characterized by excessive accumulation of triglycerides and cholesterol in lipid droplets (LDs) [15]. It is generally accepted that autophagy is activated during the early phase of NAFLD in response to the acute increase in lipid availability. Autophagy may then regulate hepatocellular lipid accumulation through selective degradation of cellular lipid stores (lipophagy). However, large or chronic lipid exposure tends to deregulate the autophagy process [13,16,17,18]. Accumulating evidence suggests that impaired autophagy prevents the clearance of LDs, damaged mitochondria, and toxic protein aggregates, which can be produced during the progression of various liver diseases, thus contributing to the development of steatosis, steatohepatitis, fibrosis, and cancer [19,20]. This supports the possibility that autophagy may be one of the key targets for the prevention and treatment of hepatic steatosis in NAFLD [21]. 

Developing a treatment for patients suffering from NAFLD is challenging because of its intricate etiology, complex diagnosis, broad spectrum of stages, and presence of concomitant disorders. However, in addition to the need for lifestyle modifications, to date, herbal medicine has emerged as an alternative approach to the prevention and/or treatment of NAFLD [22]. Certain herbal extracts and natural products are considered effective in ameliorating lipid accumulation through, at least in part, activation of autophagy. Examples include Rb2, a major ginsenoside from Panax ginseng [23]; the natural polyphenol resveratrol [24,25,26]; the bergamot polyphenol fraction (BPF), one of the dietary polyphenols [27]; capsaicin, an extract of *Capsicum annuum* and a common food supplement [28]; and many others [22]. Some natural products have already been supported by clinical studies in the treatment of NAFLD [29], such as berberine [30], resveratrol [31], and curcumin [32], found effective in improving NAFLD parameters. Thus, the literature provides valuable information on the role of natural compounds and herbal extracts as potential candidates in the management of NAFLD. 

In the light of these considerations, this work tested the ability of POE to reduce intracellular lipid accumulation in an in vitro model of hepatic steatosis using the human hepatoma cell line HepG2. The role of POE in triggering autophagy in HepG2 cells was also tested, and thus the possible correlation between POE-induced reduction of lipid accumulation and activation of an autophagic flux was verified.

## 2. Results and Discussion

### 2.1. Biochemical Composition of P. oceanica Leaf Extract (POE)

The previously developed hydroalcoholic extraction method [5] was used to recover hydrophilic compounds from *P. oceanica* leaves that were soluble enough to be readily evaluated in biological media. This method recovered 1.8 mg of dry extract per aliquot. 

POE was found to be composed mainly of polyphenols and a carbohydrate fraction. By the Folin–Ciocalteau method, POE was found to contain 3.9 ± 0.4 mg/mL of polyphenols (TP) equivalent to gallic acid, while colorimetric analysis with phenol-sulfuric acid showed that POE contains 6.0 ± 1.3 mg/mL of carbohydrates (TC) equivalent to glucose. In addition, POE exhibited radical-scavenging and antioxidant activities of 9.0 ± 0.3 mg/mL and 1.0 ± 0.2 mg/mL ascorbic acid equivalents by FRAP and DPPH assays, respectively (Table 1).

The data obtained are consistent with those obtained in previous work [6,8], supporting the efficiency and reproducibility of the extraction method.

### 2.2. Effect of POE on HepG2 Cell Viability

**Figure 2:**
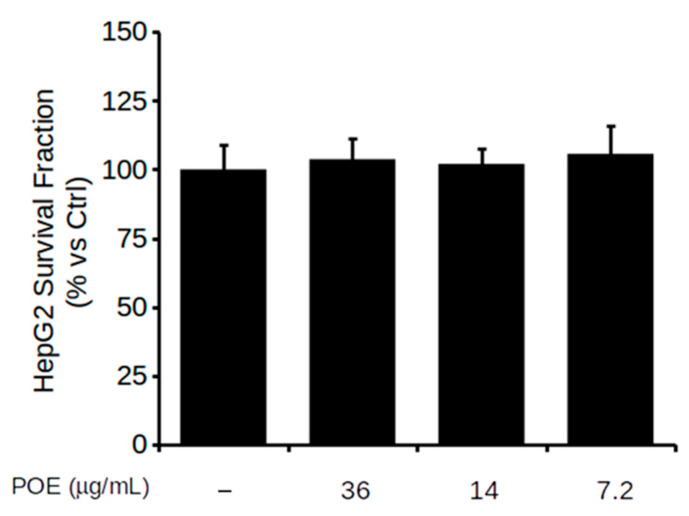
The effect of POE on HepG2 cell viability. MTT assay on cells untreated (−) or exposed to different POE concentrations for 24 h. Data were reported as the mean ± SD of at least three independent experiments.

The viability of HepG2 cells treated with POE at dilutions of 1:100, 1:250, and 1:500 (corresponding to 36, 14, and 7.2 μg/mL dry weight of extract, respectively) was assessed using MTT assay. POE treatment, ranging from 7.2 μg/mL to 36 μg/mL concentration, did not cause any reduction in cell viability, as depicted in Figure 2. Treatment with the vehicle excluded any effect of EtOH/H_2_O (70:30 *v*/*v*) on cell viability (Figure S1 in Supplementary Materials). These findings agree with the previously reported non-toxicity of POE. Indeed, the phytocomplex has already been extensively demonstrated to exert its activity without showing signs of cellular toxicity under different treatment conditions in various cell lines [5,6,7,8]. 

For subsequent experiments, POE was therefore used at the lowest dose of 7.2 μg/mL, in agreement with previous work [6,8].

### 2.3. POE Activates an Autophagic Flux in HepG2 Cells

Activation of autophagy is among the newly discovered biological properties of POE [6]. In this work, this ability of POE was verified in HepG2 cells. Autophagy pathways were explored by Western blot analysis in lysates of cells treated at different time points with POE or Rapa (used as a control for autophagy activation). 

**Figure 3:**
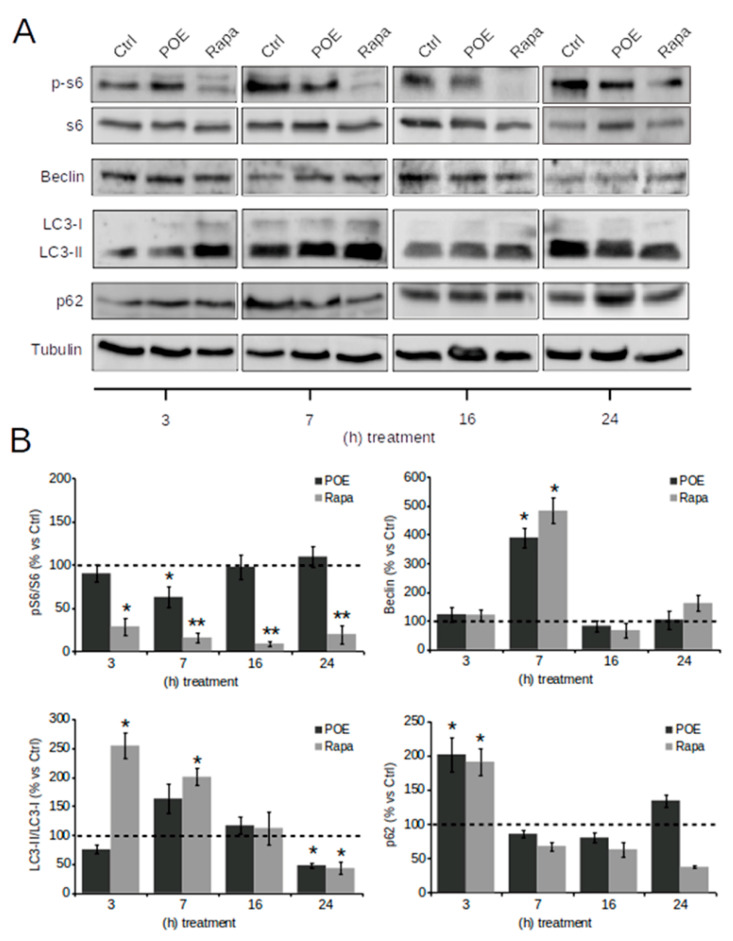
The effect of POE on autophagy flux activation in HepG2 cells. (**A**) Representative images of Western blot analysis of all the assayed protein markers of autophagy detected in HepG2 cells treated with POE (7.2 μg/mL), cells treated with Rapa (0.5 μM), or untreated cells (Ctrl). (**B**) Quantification of signals determined by densitometry analysis of at least three independent experiments. Error bars represent standard errors. * *p* < 0.05; ** *p* < 0.01 vs. untreated control cells (Ctrl; indicated by the dotted line). Kruskal–Wallis test. Pairwise comparisons were performed using Conover test.

Representative Western blot images in Figure 3A show that HepG2 cells underwent a transient autophagy flux over time induced by POE treatment, as well as cells treated by Rapa. 

Specifically, by analyzing the mammalian target of rapamycin (mTOR) signaling pathway, the best-known autophagy-suppressive regulator, it was observed that Rapa significantly reduced the levels of the phosphorylated form of S6 (p-S6) already at 3 h (28 ± 9%), maintaining them low even at 24 h (20 ± 10%) of treatment compared to untreated control cells; POE apparently caused a reduction in p-S6 levels compared with untreated control cells as early as 3 h of treatment (90 ± 9.5%)—although the data do not show statistical significance—until it resulted in a maximum reduction in p-S6 levels at 7 h of treatment (60 ± 12%). This suggests that POE contributes to an early activation of autophagy by inhibiting the mTOR signaling pathway. However, this effect ended by 16 h, when p-S6 levels returned to baseline (97 ± 14%) (Figure 3B). 

The dynamics of Beclin-1 levels were further explored, as this variation is considered a downstream event in the autophagy signaling cascade crucial for the early stage of autophagosome formation [33]. Figure 3B clearly shows that at the 7 h time point, POE caused a marked increase in Beclin-1 expression (390 ± 35%) compared to untreated control cells, supporting the observed advancement of autophagy. This effect was comparable to that induced by Rapa (483 ± 45% compared to untreated control cells). As a consequence of POE intervention on Beclin-1 expression at 7 h of treatment, the isolation membrane, a double-membrane structure encompassing cytoplasmic material, had formed to originate the autophagosome.

Because the amount of LC3-II, the phosphatidylethanolamine-conjugated form of LC3, reveals the number of autophagosomes and autophagy-related structures, LC3 is reportedly the most widely used autophagosome marker [34]. Even though LC3-II is found in the autophagosome, it is a well-known marker of autophagosome elongation. POE-treated cells exhibit a peak in LC3-II/LC3-I expression at 7 h (162 ± 15%) similarly to Rapa-treated cells (200 ± 15%) compared to untreated control cells. 

The increased expression of LC3-II further supports the evolution of autophagy in POE-treated cells. Expression levels of LC3-II/LC3-I returned to baseline from 16 h of treatment with both POE (116 ± 15%) as well as Rapa (112 ± 28%), even to the point of being down-expressed after 24 h of treatment (47 ± 5% in POE-treated cells and 42 ± 10% in Rapa-treated cells) compared to untreated control cells (Figure 3B). 

The p62 protein is a ubiquitin-binding scaffold protein that co-localizes with ubiquitinated protein aggregates in many liver pathways, which is sequestered in autophagosomes upon its direct interaction with LC3 and is selectively degraded by autophagy. Thus p62 accumulates when autophagy is inhibited and decreases when autophagy flux occurs [34]; therefore, degradation of p62 is another widely used marker to monitor autophagy activity [35].

POE caused a progressive reduction in p62 levels, starting at 7 h (85 ± 5%) until 16 h of treatment (80 ± 7%), suggesting that the autophagy process was in its final step, while Rapa treatment maintained p62 levels below baseline even until 24 h (37 ± 2%). The marked decrease in p62 after POE treatment further confirmed that POE activates an autophagy flux in HepG2 cells, with a peak activation at 7 h.

These results on the role of POE in autophagy activation with maximal efficacy at 7 h of treatment are in agreement with our previous study [6]. 

### 2.4. POE Alleviates Lipid Accumulation in HepG2 Cells by Inducing Autophagy

Autophagy is a cellular recycling mechanism essential for the maintenance of normal metabolism; impaired autophagy is often linked to the pathophysiology of many diseases. In this regard, autophagy plays a key role in lipid homeostasis through the degradation of intracellular lipid droplets (lipophagy) [17,36]. 

In this work, the effect of POE on lipid accumulation in HepG2 cells was explored in relation to its role as an inducer of autophagy. Cells were treated with POE for 24 h, while cells treated with CQ (autophagy inhibitor) and Rapa (autophagy inducer) were used as controls. 

**Figure 4:**
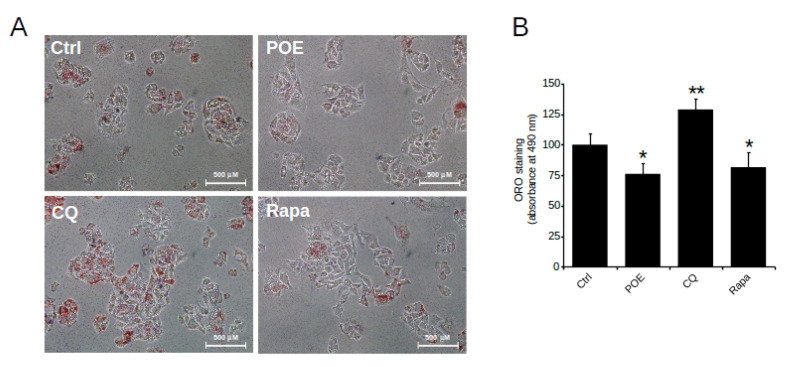
The effect of POE on lipid accumulation in HepG2 cells. (**A**) Representative image of ORO-stained HepG2 cells treated with POE (7.2 μg/mL), CQ (10 μM), and Rapa (0.5 μM) for 24 h. (**B**) Changes in intracellular lipid content assessed by measuring ORO absorbance at 490 nm. Data were reported as the mean ± SD of at least three independent experiments. * *p* < 0.05; ** *p* < 0.01 vs. untreated control cells (Ctrl). Tukey’s HSD test.

HepG2 control cells grown in complete medium were characterized by intracellular lipid accumulation, as depicted by microscope image in Figure 4A obtained after ORO staining, while vehicle treatment excluded any effect of EtOH/H_2_O (70:30 *v*/*v*) on lipid accumulation (data not shown).

Compared with untreated control cells, HepG2 cells treated with POE for 24 h showed a clear reduction in neutral lipid content (Figure 4A). Quantification of neutral lipids by solubilization of ORO with isopropanol, shown in Figure 4B, confirmed that POE caused an approximately 25% reduction in lipid accumulation (76 ± 8%) with respect to untreated control cells, suggesting a significant role of POE on lipid clearance. 

The effect of POE was comparable to that of Rapa, which resulted in an approximately 20% reduction in lipid accumulation (81 ± 11%) compared to untreated control cells (Figure 4B); in contrast, CQ-treated cells were distinguished by an increased presence of intracellular neutral lipids (128 ± 9%) compared with untreated control cells (Figure 4B). Noteworthy, differences between treatment conditions were statistically significant.

The comparable effect of POE and Rapa suggests that POE likely intervenes in lipid accumulation through activation of an autophagic flux.

Where impaired autophagy has been shown to induce hepatosteatosis [37], autophagy activation could be an effective means of maintaining normal liver function [38]. 

Some pharmacological studies have demonstrated the protective effect of natural products against excessive lipid accumulation in hepatic cells through activation of the autophagic process [22]. 

In our in vitro experiments, POE was shown to induce an autophagy flux in HepG2 cells in a time-dependent manner and to reduce intracellular lipid accumulation.

Thus, in the present study, it was tested whether POE-induced lipid elimination occurs in an autophagy-dependent manner. HepG2 cells were treated with POE for 7 h, a time point found to be relevant for POE-induced autophagy activation, and then supplemented with CQ for up to 24 h (POE + CQ). 

**Figure 5:**
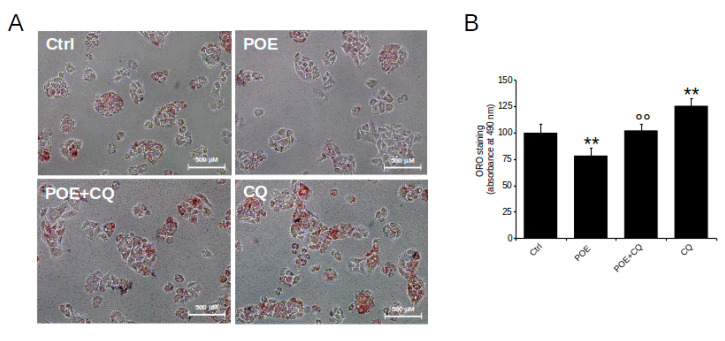
POE reduces lipid accumulation in HepG2 cells through autophagy activation. (**A**) Representative image of ORO-stained HepG2 cells treated with POE (7.2 μg/mL), CQ (10 μM), or POE added with CQ at 7 h (POE + CQ) until 24 h. (**B**) Changes in intracellular lipid content assessed by measuring ORO absorbance at 490 nm. Data were reported as the mean ± SD of at least three independent experiments. ** *p* < 0.01 vs. untreated control cells (Ctrl); °° *p* < 0.01 vs. POE-treated cells. Tukey’s HSD test.

The microscopy image shown in Figure 5A depicts a clear increase in lipid accumulation in POE + CQ cells compared with POE-treated cells. Quantification of intracellular neutral lipids by solubilization of ORO with isopropanol, plotted in Figure 5B, confirmed that lipid accumulation in POE + CQ cells increased by approximately 30% (102 ± 6%) compared to cells treated with POE alone (78 ± 7.7%). 

Recently, it has become known that autophagy mediates the elimination of stored lipid droplets [39]. Our data indicate that in the presence of CQ, the ability of autophagosomes to fuse with lysosomes is reduced, resulting in the accumulation of intracellular lipids; the altered autophagy flux provides a possible reason for the restoration of higher basal intracellular lipid levels in POE + CQ cells. This suggests that the effect of the POE phytocomplex on lipid accumulation is completely reversed by CQ-induced autophagy blockade.

Overall, this study provides evidence of the potential of POE to alleviate intracellular lipid accumulation through activation of autophagy. Thus, POE, which can control this process in liver cells, could have promising potential as an alternative medical strategy for a variety of disease conditions, including NAFLD [22,40]. 

## 3. Materials and Methods

### 3.1. Chemicals

Dulbecco’s Modified Eagle’s Medium (high-glucose DMEM-HG), fetal bovine serum (FBS), L-glutamine, penicillin and streptomycin, 1-(4,5-dimethylthiazol-2-yl)-3,5-diphenyl formazan (MTT), Oil red O (ORO), chloroquine (CQ), and rapamycin (Rapa) and all chemicals and solvents were purchased from Merck KGaA (Darmstadt, DA, Germany). Electrophoresis reagents were purchased from Bio-Rad Laboratories (Hercules, CA, USA). Primary antibodies were provided by Cell Signaling Technology (Beverly, MA, USA), Molecular Probes^TM^ (Invitrogen, Carlsbad, CA, USA), and Abcam (Cambridge, UK) (Table 2). Anti-mouse IgG HRP-linked and anti-rabbit IgG HRP-linked secondary antibodies were obtained from Molecular Probes^TM^ (Invitrogen, Carlsbad, CA, USA). Disposable plastics were from Sarstedt (Nümbrecht, Germany). 

The leaves of *P. oceanica* were collected in July 2020 from the protected area of Meloria by personnel of the Interuniversity Center of Marine Biology and Applied Ecology G. Bacci (CIBM, Livorno, Tuscany, Italy) at a depth of about 15 m at the following geographical coordinates: 43° 35′ 13″ N and 010° 10′ 21″ E.

The CIBM was authorized for the collection of *P. oceanica* leaves by the Direction of Regional Parc of Migliarino, San Rossore, Massaciuccoli, formerly the managing institution of the Marine Protected Area (MPA) “Secche della Meloria.” The following permission “Ente Parco Reg. M.S.R.M. Prot. 0012275 del 20-11-2019 partenza Cat. 7 Cl. 7 SCI.8” authorized the CIBM for institutional and research activities (including monitoring and sampling of both water and biota) inside all the MPA from 20 November 2018 to 31 December 2020.

### 3.2. Preparation of P. oceanica Extract

The collected leaves were removed from the epiphytes and carefully washed with bi-distilled water. The hydrophilic component was recovered according to the method previously described [5]. Briefly, 1 g of *P. oceanica* dried leaves were minced and suspended overnight in 10 mL of EtOH/H_2_O (70:30 *v*/*v*) at 37 °C under stirring and subsequently at 65 °C for 3 h. The hydroalcoholic extract was then separated from the debris by centrifugation at 2000× *g*, and the recovered supernatant was mixed with n-hexane in a 1:1 ratio. The hydrophilic phase of the extract was recovered after vigorous agitation in a separating funnel, dispensed into 1 mL aliquots, and then dried using a Univapo^TM^ vacuum concentrator. Each aliquot of *P. oceanica* leaf extract (1.8 mg of dry extract) was then dissolved in 0.5 mL of 70% (*v*/*v*) ethanol prior to use and hereafter referred to as POE.

### 3.3. Determination of Total Polyphenols and Carbohydrates

Total polyphenol (TP) and total carbohydrate (TC) content in POE was determined using the colorimetric Folin–Ciocalteau’s method and phenol-sulfuric acid colorimetric methods, respectively [5]. Gallic acid (0.5 mg/mL) and D-glucose (1 mg/mL) were used as a reference in the range of 0–10 mg and 0–50 mg, respectively, to determine TP and TC values. 

TP and TC were expressed as milligrams of gallic acid and D-glucose equivalents, respectively, per milliliter of extract after resuspension. 

### 3.4. Antioxidant Assays

The antioxidant and radical-scavenging activities of POE were investigated using ferric-reducing/antioxidant power (FRAP) assay and α,α-diphenyl-β-picrylhydrazyl (DPPH) assay, respectively [5]. Ascorbic acid (0.1 mg/mL) was used as a reference in the range of 0–4 mg to evaluate both activities. The antioxidant and radical-scavenging activities of POE were expressed as milligrams of ascorbic acid equivalents per milliliter of extract after resuspension.

### 3.5. Cell Line and Culture Conditions

The human hepatoma cell line (HepG2), purchased from the American Type Culture Collection (ATCC^®^, HB-8065TM), was grown in a humidified atmosphere of 5% CO_2_ at 37 °C in DMEM-HG supplemented with 10% FBS, 100 μg/mL streptomycin, 100 U/mL penicillin, and 2 mM L-glutamine (complete medium). At 90% confluence, cells were collected by scraping and seeded at an appropriate cell density.

### 3.6. Cell Viability Assay

Cell viability was determined by MTT assay. Cells were grown in 96-well plates (3 × 10^4^ cells/well) for 24 h in complete medium. Next, cells were treated with 1:100, 1:250, and 1:500 dilutions of POE (corresponding to 36, 14, and 7.2 μg/mL dried weight of extract, respectively) for 24 h. Cells treated with the EtOH/H_2_O (70:30 *v*/*v*) vehicle and untreated cells were used as controls.

After cell treatments, 100 μL of MTT solution (0.5 mg/mL) was added to each well and cells were incubated in the dark at 37 °C for a further 1 h. After washing out the supernatant, the insoluble formazan product was dissolved in 100 μL/well of dimethyl sulfoxide (DMSO). Absorbance values were measured using a iMARK microplate reader (Bio-Rad Laboratories, USA) at 595 nm. Data were expressed in terms of percentages with respect to untreated control cells.

### 3.7. Western Blot Analysis

HepG2 cells (2 × 10^5^ cells/well) were seeded in 35 mm dishes in complete medium and incubated overnight. To study the role of POE in the activation of an autophagic flux, cells were treated with POE (7.2 μg/mL) for different time points, from 3 h to 24 h. Cells treated with Rapa (0.5 μM) were used as an autophagy activation control. After treatments, cells were washed with PBS and then lysed in 80 μL of Laemmli buffer (62.5 mM Tris-HCl pH 6.8, 10% (*w*/*v*) SDS, 25% (*w*/*v*) glycerol) without bromophenol blue. Whole-cell lysates were collected and boiled at 95 °C for 5 min and centrifuged to remove cell debris (12,000× *g* for 5 min at 4 °C). The BCA protein assay kit (Bio-Rad Laboratories, Hercules, CA, USA) was used to measure protein levels. Briefly, 25 μg of proteins from each sample, added with β-mercaptoethanol and bromophenol blue, were electrophoresed by 12% SDS-PAGE and transferred to PVDF membranes (0.45 μm). The membranes were incubated with blocking solution (5% (*w*/*v*) BSA in 0.1% (*v*/*v*) PBS-Tween^®^-20) for 1 h at room temperature. Membrane incubation with the desired primary antibody at appropriate dilution was conducted overnight at 4 °C (Table 1). 

After three washes in 0.1% (*v*/*v*) PBS-Tween^®^-20 solution, the membranes were incubated for 1 h at room temperature with a specific secondary antibody (goat anti-rabbit IgG or goat anti-mouse IgG) diluted 1:10,000 in blocking solution. The membranes were finally washed three times in 0.5% (*v*/*v*) PBS-Tween^®^-20 before being stained with Clarity Western ECL solution. Signal of chemiluminescence were acquired with an Amersham^TM^ 600 Imager imaging system (GE Healthcare Life Science, Pittsburgh, PA, USA). Quantity One software (Bio-Rad Laboratories) was used for densitometric analysis.

### 3.8. ORO Staining

HepG2 cells were seeded (6 × 10^4^ cells/well) in 24-well plates overnight and then treated with POE (7.2 μg/mL) for 24 h. To test the impact of the autophagy process on lipid accumulation, cells were also exposed to CQ (10 μM) and Rapa (0.5 μM) treatment. Following this, the cells were washed with PBS and fixed for 10 min in 2% (*v*/*v*) paraformaldehyde. Subsequently, cells were washed twice with PBS and left to dry completely. Neutral lipids were stained for 30 min at 37 °C with 200 μL/well of Oil red O working solution (60% in distilled water). Excess dye was washed away with distilled water until the water no longer had a visible pink color. After the wells had completely dried, the stained lipid droplets in cells were examined and photographed under a Nikon TS-100 microscope equipped with a digital acquisition system (Nikon Digital Sight DS Fi-1; Nikon, Minato-ku, Tokyo, Japan). Finally, cellular lipid accumulation was measured by adding 200 μL/well of isopropanol. Absorption was measured at 490 nm using an iMARK microplate reader (Bio-Rad Laboratories, USA).

### 3.9. Statistical Analysis

Where not otherwise specified, data are expressed as the mean ± SD of at least three independent experiments.

For ORO and MTT experiments, signals acquired from independent experiments were normalized by mean centering (i.e., each replicate measurement was divided by the mean of the replicates in order to compensate for batch experimental fluctuations) and differences were assessed by one-way ANOVA followed by Tukey’s HSD test after normality check with the Shapiro–Wilk test. 

For Western blotting, difference between house-keeping-normalized intensity signals were assessed by the Kruskall–Wallis test, followed by the Conover post hoc test.

Statistical differences were called at *p* ≤ 0.05.

## 4. Conclusions

In summary, this study revealed that POE protects against lipid accumulation in HepG2 cells by promoting autophagy through inhibition of the mTOR pathway. 

*P. oceanica* is a marine plant with several bioactive properties, including antidiabetic and anti-inflammatory effects [41]. Thus, the possibility that *P. oceanica* may hit multiple pathogenic liver disease targets—lowering blood sugar, inhibiting the inflammatory state, and blocking the reduction in hepatic lipid accumulation—makes this traditional herbal remedy a potential effective weapon against the prevention and/or treatment of steatosis and related disease conditions. Indeed, NAFLD is a metabolic condition commonly associated with type 2 diabetes mellitus and often combined with a chronic inflammatory state. Given the lack of approved and recognized drug therapies for NAFLD, this study offers new insights into the mechanisms of action of *P. oceanica* that are a first step for further in vitro and in vivo studies in order to identify alternative and complementary strategies for disease management. 

However, because this study was performed on the HepG2 cell line, it is essential that the data also be validated on primary hepatocytes in future studies. In addition, studies in in vivo models are absolutely necessary to test the applicability of *P. oceanica* in the management of NAFLD.

## Figures and Tables

**Table 1:** Polyphenol and carbohydrate content in POE and its antioxidant and radical-scavenging properties. All values (mg/mL) are reported as means ± SD of at least three independent extractions.

	TP	TC	Antioxidant	Radical Scavenging
Method	Folin–Ciocalteau	Phenol/sulfuric acid	Ferrozine^®^	DPPH
Reference control	Gallic acid	Glucose	Ascorbic acid	Ascorbic acid
POE	3.9 ± 0.4	6.0 ± 1.3	1.0 ± 0.2	9.0 ± 0.3

**Table 2:** Specifications of primary antibodies used in Western blotting experiments.

Primary Antibody	Target	Dilution	Host	Source	Lot
SQTSM1/p62	SQTSM1/p62 protein	1:1000	Rabbit	Abcam	#GR84445-1
LC3	Microtubule-associated protein light chain 3	1:1000	Rabbit	Invitrogen	#UD2753807C
Beclin-1	Beclin-1 protein	1:1000	Rabbit	Cell Signaling	#6
S6	Ribosomal protein S6	1:1000	Rabbit	Cell Signaling	#7
p-S6	Ribosomal protein S6 (Ser235/236)	1:2000	Rabbit	Cell Signaling	#16
α-Tubulin	α-Tubulin protein	1:1000	Mouse	Genetex	#43922

## Data Availability

All data has been present in main text and supplementary materials.

## Supplementary Materials

The following are available online at www.mdpi.com/article/10.3390/ph14100969/s1, Figure S1. The effect of EtOH/H_2_O vehicle (70:30 *v/v*) on the viability of HepG2 cells. MTT assay on untreated cells (-) or exposed to different dilutions of EtOH/H_2_O (70:30 *v/v*) for 24 h. The amount of EtOH/H_2_O (70:30 *v/v*) applied corresponds exactly to that used in POE cell treatment. Data were reported as mean ± SD of three independent experiments.
