# Supplementary material for: Posidonia oceanica (L.) Delile Extract Reduces Lipid Accumulation through Autophagy Activation in HepG2 Cells
Source: Pharmaceuticals (Basel). 2021 Sep 24;14(10):969. doi: 10.3390/ph14100969 (PMC8540819; doi:10.3390/ph14100969)
Supplement: Supplementary file 1 [file pharmaceuticals-14-00969-s001.zip › pharmaceuticals-1321315-supplementary.pdf]

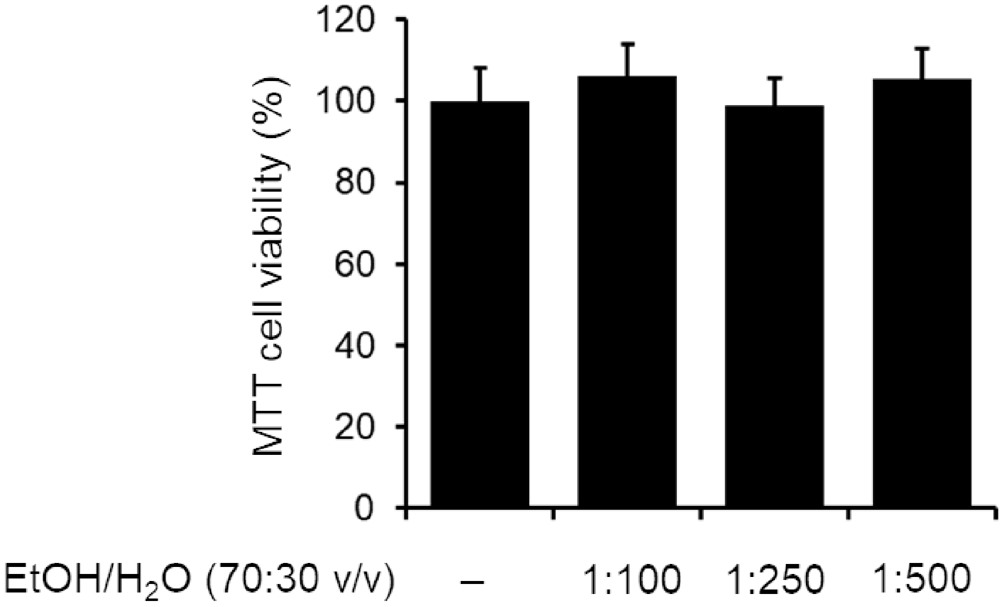

Figure S1. The effect of EtOH/H_2O vehicle (70:30 v/v) on the viability of HepG2 cells. MTT assay on untreated cells (-) or exposed to different dilutions of EtOH/H_2O (70:30 v/v) for 24h. The amount of EtOH/H_2O (70:30 v/v) applied corresponds exactly to that used in POE cell treatment. Data were reported as mean ± SD of three independent experiments.
